# EHMTI-0060. Neurosarcoidosis and chronic headache - case report

**DOI:** 10.1186/1129-2377-15-S1-C63

**Published:** 2014-09-18

**Authors:** Z Vukojevic

**Affiliations:** 1 Department of Neurology, Clinical centre, Banja Luka, Bosnia and Herzegovina

## Introduction

Sarcoidosis is a granulomatous disease of unknown etiology that in 5% of patients affects the central nervous system. Headache is one of the most common neurological symptoms.

## Case report

A 40-years old female patient gradually devoleped diffuse headache over period of six months. As headache become more severe, analgesics were of little or no help. During the last two months she gaind hearing loss, gait instability and mild weaknesses in lower extremities, that were detected during neurological assessement.

Five years prior to hospitalization patient was treated for pulmonary and lymph node sarcoidosis. The diagnosis was confirmed by cytological finding after parotid gland puncture and CT of the chest. She was treated with prednisone for ten months and she was fully recovered.

MRI and MR spectroscopy of the brain: non-specific granulomatous inflammation in the pons (15x12x14 mm). Cerebrospinal fluid: proteinorachia, hypoglycorrhachia, mild pleocytosis.

She was treated with methylprednisolone 1000 mg/day for 5 days and then switched to 60 mg of prednisone daily. After two weeks she was free from severe headache, after two months hearing and balance were significantly improved, steroids were gradually reduced and after 6 months excluded.

MRI of the brain (check-up): after two months granulomatous changes in the pons is reduced (8x5x6 mm) and after six months finding is normal.

## Conclusion

Neurosarcoidosis is a rare cause of chronic headache which responds poorly to analgesics, the diagnosis is often easily overlooked, and treatment must be vigorous with pulse doses of steroids and oral steroids.

No conflict of interest.

